# Animal and Plant Protein Intake and Body Mass Index and Waist Circumference in a Korean Elderly Population

**DOI:** 10.3390/nu10050577

**Published:** 2018-05-08

**Authors:** Ki-Byeong Park, Hyun Ah Park, Jae-Heon Kang, Kyoungwoo Kim, Young Gyu Cho, Jinyoung Jang

**Affiliations:** Department of Family Medicine, Seoul Paik Hospital, Inje University College of Medicine, Seoul 100032, Korea; kipyong@hanmail.net (K.-B.P.); fmleader@nuri.net (J.-H.K.); kwkimfm@gmail.com (K.K.); jacobel@hanmail.net (Y.G.C.); patchjang@gmail.com (J.J.)

**Keywords:** animal protein, plant protein, elderly, obesity, glomerular filtration rate

## Abstract

Controversy exists on whether animal and plant proteins influence obesity differently. The purpose of this study was to evaluate the association between total, animal, and plant protein intake with the body mass index (BMI), waist circumference (WC), and renal function in the Korean elderly. Study participants included Korean adults aged 60 years or older from the Korean National Health and Nutrition Examination Survey in 2013–2014. Height, weight, and waist circumference were measured and the body mass index was calculated. One-day 24-hour recall data were used to estimate daily total, animal, and plant protein intake. Glomerular filtration rate (GFR) was calculated by using the Modification of Diet in Renal Disease (MDRD) equation. General linear modellings were used to assess the relationships between protein intake, BMI and WC. The mean age was 69.2 ± 0.2 years and 44.2% were male. The total daily protein intake was 1.1 ± 0.02 g/kg/day and 0.9 ± 0.02 g/kg/day for males and females, respectively. Only one third of protein intake was from animal sources. In males, BMI (regression coefficient (95% CI); −1.30 (−1.55, −1.06), *p* < 0.001; −0.29 (−0.52, −0.05), *p* = 0.016; −1.30 (−1.8, −1.02), *p* < 0.001, respectively) and WC (−3.87 (−4.58, −3.16), *p* < 0.001; −0.90 (−1.58, −0.22), *p* = 0.010; −3.88 (−4.68, −3.08), *p* < 0.001, respectively) decreased as daily intake of plant protein (g/kg/day), animal protein (g/kg/day) and total protein (g/kg/day) increased. Similar associations were shown in Korean females. GFR was not associated with protein intake regardless of protein source in both sexes. In Korean adults aged 60 years or older, the protein intake was associated with a favorable obesity index without decrease in renal function. The effect was similar in both males and females, with both animal and plant proteins.

## 1. Introduction

Overweight and obesity are rising in Korea [1]. In particular, the prevalence of obesity among people aged 70 and over rose sharply from 31.5% in 2005 to 37.4% in 2015 [2]. Obesity not only increases the risk of metabolic diseases, but also increases the all-cause mortality rate in the elderly population [3,4]. Therefore, measures to contain the epidemic of obesity in the elderly population are needed.

The association between obesity and protein intake has become the topic of interest. Observational studies in the US have reported that protein intake above the Recommended Daily Allowance (RDA) reduces body weight and waist circumference (WC) [5], and improves body composition [6]. In addition, a longitudinal study of the elderly showed that the increase in protein intake decreases the risk of sarcopenia and obesity [7]. These associations were observed across all age groups.

Whether the effect of protein on obesity depends on the protein source, i.e., plant versus animal source, is unclear. A study done in the United States showed that the increased consumption of both plant and animal protein improved body mass index (BMI) and WC [8]. However, Belgian research showed that only plant protein significantly improved the obesity indexes [9].

Recent Korean health statistics showed that the only age group in Korea that consumes dietary protein less than the RDA is the elderly population [10]. The daily total protein intake of Korean males and females in their 70s were 61.4 ± 1.4 g/day and 45.4 ± 1.0 g/day, respectively, and it was 52.2 ± 2.4 g/day and 39.5 ± 1.5 g/day for persons over 80 years old [11]. Furthermore, because the Koreans eat rice as staple food, more than two-thirds of protein intake is taken as a plant protein [11]. By contrast, in the United States, the daily protein intake was 80.8 ± 2.0 g/day and 60.0 ± 1.5 g/day for males and females over 70 years old, respectively, while consuming more than 60% of the total protein intake from animal sources [12,13]. Due to differences in the quantity and quality of protein intake between Korea and the west, study results from western countries may not be directly applied to the Korean population.

Only few studies investigated the relationship between protein and obesity in East Asians. Therefore, we sought to evaluate the relationship between the protein intake and obesity according to protein source (animal versus plant protein) in the elderly Korean population using the nation-wide representative sample of Koreans. We also evaluated the association between protein intake and renal function.

## 2. Materials and Methods

### 2.1. Study Participants

The Korea National Health and Nutrition Examination Survey (KNHANES) is a population-based cross-sectional survey conducted to assess the health-related behavior, the health condition, and the nutritional state of Koreans. The KNHANES consists of health interview surveys (household survey, comorbidity, periodic health examination, quality of life, injury, health care utilization, education and economic activities, and health behavior survey), health examination surveys and nutrition surveys (dietary behavior, food frequency questionnaire, and 24-hour dietary recall). Detailed descriptions of the plan and operation of the survey have been described on the KNHANES website (http://knhanes.cdc.go.kr/). In 2013 and 2014, participation rate of health interview survey and health examination survey were 75.0% and 73.9%, respectively, and that of nutrition survey was 82.7% and 81.7%, respectively.

Our study subjects included a total of 2549 persons aged 60 years or older (male: 1127; female 1427) who participated in all three surveys from the 2013~2014 KNHANES. We excluded participants who reported to consume <500 kcal or >5000 kcal a day or those with missing data on the health behavior survey of the health interview survey. The study protocol was approved by the Institutional Review Board of Seoul Paik Hospital (IRB No. 2017-11-010). Informed consent was waived by the Institutional Review Board.

### 2.2. Covariate Measurements

We collected data on the demographic (age and sex) and socioeconomic (household income and education) factors. Participants were categorized according to age (60 to 69 years, 70 to 79 years, ≥80 years), household income (upper, upper middle, lower middle and lower) and education level (<9 years, 10–12 years, and ≥13 years).

Data on comorbidities including hypertension, dyslipidemia, stroke, ischemic heart disease, osteoarthritis, rheumatoid arthritis, asthma, diabetes, thyroid disease, chronic kidney disease, chronic liver disease or any type of cancer were collected.

### 2.3. Health Behavior Measurement

Smoking status was divided into smokers and non-smokers. Alcohol intake was divided into 0, 1, and ≥2 times per week. The level of physical activity was assessed by calculating the Metabolic Equivalent for Task (MET) per week using the International Physical Activity Questionnaire (IPAQ) which consists of self-reported exercise days per week and exercise duration of walking, moderate-intensity and vigorous-intensity exercise [14].

### 2.4. Body Mass Index and Waist Circumference

Height (SECA 225; Seca, Hamburg, Germany) and weight (GL-6000-20; CAS, Yangju-si, Korea) were measured while the participant wore a lightweight gown or underwear. BMI was calculated and classified into ≤18.4 kg/m^2^ (underweight), 18.5–22.9 kg/m^2^ (normal), 23.0–24.9 kg/m^2^ (overweight), and ≥25.0 kg/m^2^ (obesity) according to the Asia Pacific Standards of the WHO-recommended definition of Obesity [15].

The WC measured by a well-trained examiner to the nearest 0.1 cm at the mid-point between the lower rib and the pelvic iliac crest.

### 2.5. Assessment of Protein and Other Macronutrient Intake

One-day 24-h recall data were used to estimate the daily protein and macronutrient intake. Each food consumed was classified into 19 food groups adapted by the KNHANES [16]. Of the 19 food groups, proteins taken from grains, potatoes, sugars, beans and legumes, nuts, plants, mushrooms, fruits, seaweeds, drinks and alcohols, condiments, and other (plant) were classified as plant protein and those taken from meat, eggs, fish and shellfish, dairy foods, oil (animal), and other (animal) were classified as animal protein. Protein intake was further quantified (1) as protein intake in grams per day; (2) percentage of energy from protein; and (3) grams per kilogram body weight.

### 2.6. Renal Function Assessment

Glomerular filtration rate (GFR) was calculated according to Modification of Diet in Renal Disease-GFR (MDRD-GFR) formula [17].

### 2.7. Statistical Analysis

All analyses were stratified according to gender. Descriptive statistics were presented as means or proportions with their standard errors. The trend test was used to evaluate the relationship between total protein intake quartiles and macronutrients intake, animal and plant protein intake, anthropometric measurements, and physical activity level.

Multivariate general linear modellings were carried out to examine the relationship between protein intake quartiles and obesity index measured by BMI, WC and renal function index measured by serum creatinine and GFR. Adjusted means of BMI and WC by protein intake quartiles were presented after controlling age (year), household income quartiles, education (≤9 years, 10–12 years, 13≤ years), presence of chronic disease (yes or no), current smoking status (yes or no), alcohol intake frequency per week (0, 1, 2≤), physical activity (MET/week), % energy from fat (%), % energy from carbohydrate (%) and total energy intake (kcal). For the modelling of serum creatinine and GFR, age (year), BMI (kg/m^2^), physical activity (MET/week), household income quartiles, current smoking status (yes or no), and alcohol intake frequency per week (0, 1, 2≤) were controlled.

A two-sided probability value <0.05 was considered to indicate a statistically significant difference. Statistical tests were performed using SPSS 18 statistical package (SPSS Inc., Chicago, IL, USA) incorporating sampling weight while considering the multistage probability sampling design of KNHANES and the nonresponses.

## 3. Results

### 3.1. Study Population

A total of 2549 participants were enrolled. The mean age was 69.5 ± 0.2 years; 44% were male (Table 1), two-thirds had lower or middle lower household income and education less than 9 years; 77% had at least one comorbidity.

The proportion of overweight or obese participants were 55.8% and 64.0%, the mean waist circumference 84.8 ± 0.3 cm and 82.5 ± 0.3 cm, and the physical activity level measured by MET were 2275.1 ± 92.8 MET/week and 1542.6 ± 66.3 MET/week, for males and females, respectively.

### 3.2. Characteristics of Study Participants According to Protein Intake

Total protein intake for males was 67.1 ± 1.1 g/day, accounting for 13.1 ± 0.1% of the total energy intake (Table 2). Total protein intake per body weight was 1.1 ± 0.02 g/kg/day, of which 0.6 ± 0.01 g/kg/day was from plant sources and 0.4 ± 0.02 g/kg/day was from animal sources, thus the proportion of animal protein to the total protein was 33.4 ± 0.7%. Females reported lower total protein intake (0.9 ± 0.02 g/kg/day) and a lower proportion of animal to total protein (29.0 ± 0.7%) than males. Plant protein intake was the main contributor to the total protein intakes in both sexes. The energy % from carbohydrate (*p* < 0.001) decreased, and that from fat (*p* < 0.001) increased in both sexes as the quartiles of total protein intake (g/kg/day) increased.

In males, total protein intake was positively associated with both plant (*p* < 0.001) and animal (*p* < 0.001) protein intake. From the lowest to highest quartiles of total protein intake, the plant protein intake approximately doubled from 28.0 ± 0.5 g/day to 52.7 ± 1.3 g/day while animal protein increased six times from 8.1 ± 0.4 g/day to 53.4 ± 2.5 g/day. Similar patterns were observed in females. The increase in animal protein intake, rather than plant protein intake contributed more to the increase of total protein intake.

### 3.3. Body Mass Index, Waist Circumference and Protein Intake

**Figure 1:**
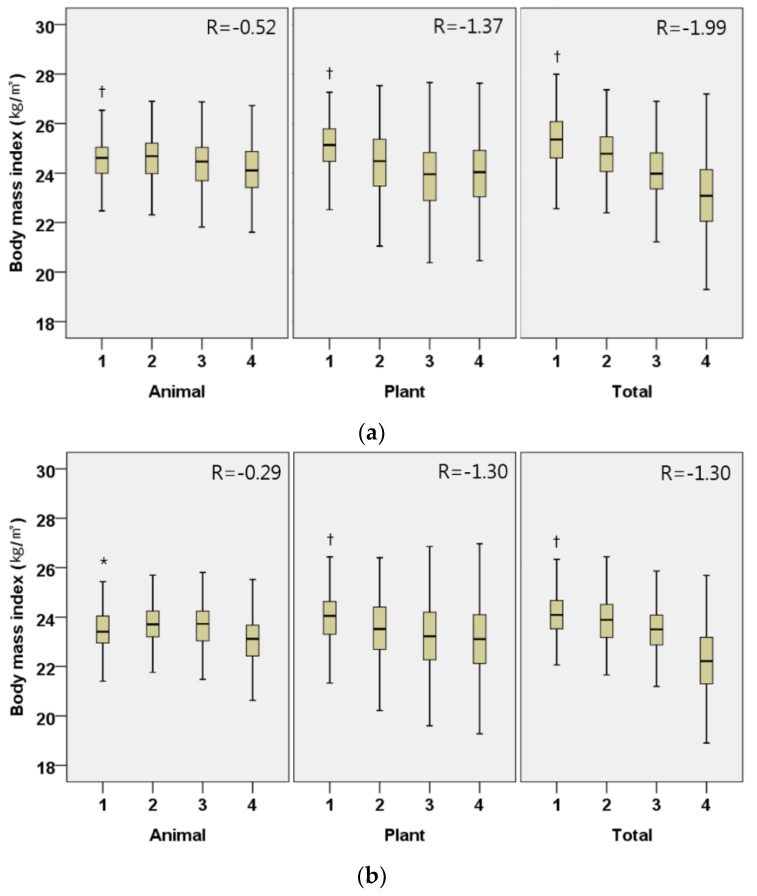
Adjusted body mass index (kg/m^2^) by the quartiles of daily animal, plant, and total protein intake per weight (g/kg/day) in Korean elderly population. (**a**) Male; (**b**) Female. All models are adjusted for age (year), household income quartile, education (≤9 years, 10–12 years, 13≤ years), presence of chronic disease (yes or no), current smoking status (yes or no), alcohol intake frequency per week (0, 1, 2≤), physical activity (MET/week), % energy from fat (%), % energy from carbohydrate (%) and total energy intake (kcal). Participants were divided into quartiles for daily animal, plant, and total protein intake per weight (g/kg/day); male animal (Q1 < 0.13, Q2 0.13–0.29, Q3 0.30–0.56, Q4 0.56<), male plant (Q1 < 0.45, Q2 0.45–0.61, Q3 0.62–0.79, Q4 0.79<), male total (Q1 < 0.70, Q2 0.70–0.97, Q3 0.98–1.30, Q4 1.30<), female animal (Q1 < 0.07, Q2 0.07–0.21, Q3 0.21–0.40, Q4 0.40<), female plant (Q1 < 0.42, Q2 0.42–0.57, Q3 0.58–0.74, Q4 0.74<), female total (Q1 < 0.59, Q2 0.59–0.82, Q3 0.83–1.14, Q4 1.14<); β-coefficient (95% confidence interval) male animal −0.29 (−0.52, −0.05), male plant −1.30 (−1.55, −1.06), male total −1.30 (−1.58, −1.02), female animal −0.52 (−0.75, −0.30), female plant −1.37 (−1.60, −1.14), female total −1.99 (−2.29, −1.70); * *p* < 0.05, ^†^ *p* < 0.001 by trend test.

**Figure 2:**
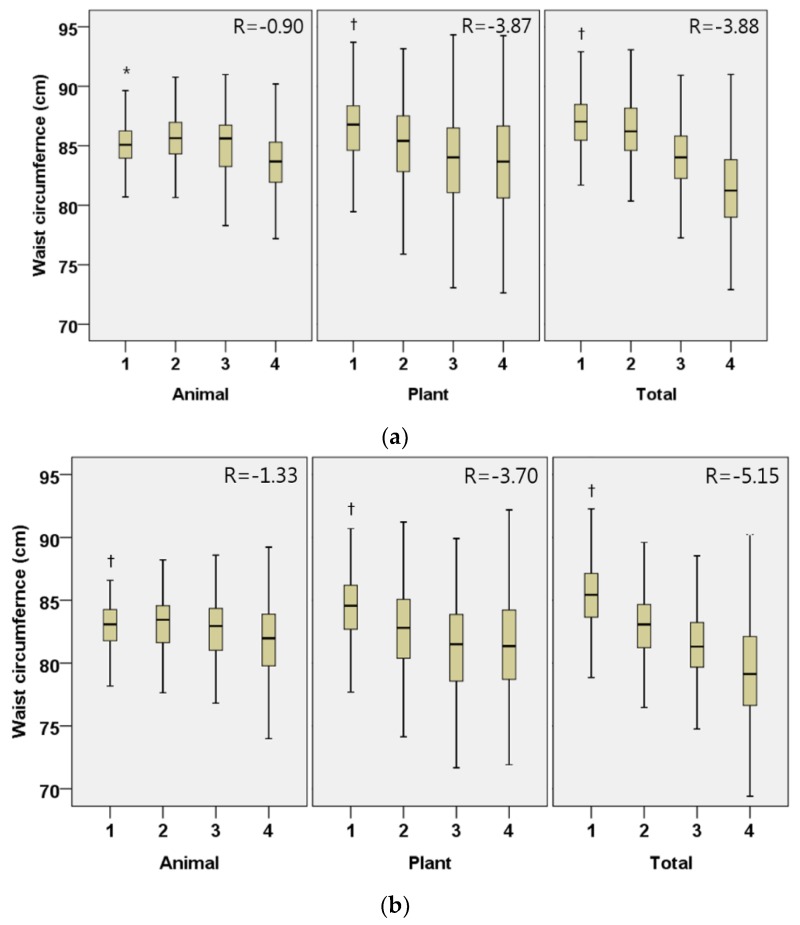
Adjusted waist circumference by the quartiles of daily animal, plant, and total protein intake per weight (g/kg/day) in Korean elderly population. (**a**) Male; (**b**) Female. All models are adjusted for age (year), household income quartile, education (≤9 years, 10-12 years, 13≤ years), presence of chronic disease (yes or no), current smoking status (yes or no), alcohol intake frequency per week (0, 1, 2≤), physical activity (MET/week), % energy from fat (%), % energy from carbohydrate (%) and total energy intake (kcal). Participants were divided into quartiles for daily animal, plant, and total protein intake per weight (g/kg/day); male animal (Q1 < 0.13, Q2 0.13–0.29, Q3 0.30–0.56, Q4 0.56<), male plant (Q1 < 0.45, Q2 0.45–0.61, Q3 0.62–0.79, Q4 0.79<), male total (Q1 < 0.70, Q2 0.70–0.97, Q3 0.98–1.30, Q4 1.30<), female animal (Q1 < 0.07, Q2 0.07–0.21, Q3 0.21–0.40, Q4 0.40<), female plant (Q1 < 0.42, Q2 0.42–0.57, Q3 0.58–0.74, Q4 0.74<), female total (Q1 < 0.59, Q2 0.59–0.82, Q3 0.83–1.14, Q4 1.14<); β-coefficient (95% confidence interval) male animal −0.90 (−1.58, −0.22), male plant −3.87 (−4.58, −3.16), male total −3.88 (−4.68, −3.08), female animal −1.33 (−1.95, −0.71), female plant −3.70 (−4.42, −2.99), female total −5.15 (−5.93, −4.37); * *p* < 0.05, ^†^ *p* < 0.001 by trend test.

In males, the daily intake of plant protein (regression coefficient (95% CI); −1.30 (−1.55, −1.06), *p* < 0.001; −3.86 (−4.58, −3.16), *p* < 0.001, respectively), animal protein (−0.29 (−0.52, −0.05), *p* = 0.016; −0.90 (−1.58, −0.22), *p* = 0.010) and total protein (−1.30 (−1.58, −1.02), *p* < 0.001; −3.88 (−4.68, −3.08), *p* < 0.001) were all inversely associated with BMI and WC, after adjusting for covariates (Figure 1 and Figure 2). The associations between protein intake and obesity index were more marked in plant protein than in animal protein. Similar associations were shown in Korean females.

### 3.4. Markers of Kidney Function and Protein Intake

To explore the relationship between protein intake and renal function, we excluded 18 participants who reported physician-diagnosed renal disease and 311 patients who had missing data on serum creatinine, so that the data of 2220 participants (1007 males and 1213 females) were included in this analysis (Table 3). None of plant protein, animal protein, and total protein intake had significant relationship with serum creatinine or GFR in both sexes.

## 4. Discussion

There is controversy of whether animal and plant proteins have different effects on obesity. In this study, a representative sample of Koreans aged 60 years or older revealed that animal and plant protein intakes, as well as the total protein intake, had negative associations with BMI and WC.

### 4.1. Total Protein, Plant Protein, Animal Protein and Obesity

Though there has been some consensus on the beneficial effect of protein intake on obesity indexes such as BMI and WC, there has been inconsistent results on whether the source of protein, i.e., plant or animal, has different effects on obesity [5,6,8,18]. Previous studies showed that protein intake from plant sources improved obesity index in cross-sectional and longitudinal studies. Lin et al. [9] reported that plant protein intake was inversely correlated with BMI and WC in Belgian adults, using two non-consecutive 24-h recall. Deibert et al. [19] implemented a randomized trial that provided a substitution diet containing high-soy-protein and reported that in overweight and obese subjects, body weight and BMI decreased as soy protein intake increased. In addition, our study found a negative association between plant protein intake and the obesity index.

Nonetheless, the effect of animal protein intake on obesity showed conflicting results. Berryman et al. [8] developed the usual protein intake from the US NHANES with the use of the National Cancer Institute method [20] and reported that animal protein intake was negatively associated with the risk of obesity and abdominal obesity. By contrast, Bujnowski et al. [21] obtained crossed-checked dietary information for three consecutive days using Burke’s comprehensive dietary history method [22] and reported that obesity risk increased with an increase in animal protein intake during a 7-year longitudinal study. Alkerwi et al. [23] also reported that the risk of abdominal obesity increases with increasing intake of meat, fish and fish products, using 134-item semiquantitative food frequency questionnaire. In our study, it was found that animal protein intake correlated negatively with BMI and WC.

The reason for inconsistent results regarding the intake of animal protein is unclear. Possible explanations are differences in culinary culture among countries leading to different quality and quantity of protein intake. Studies whose participants had a high daily protein intake tend to show positive associations between protein intake and obesity while those with a low protein intake tend to display negative associations. For example, the mean intake of the lowest quartile of animal protein in the study from Bujnowski et al. [21] was 74.7 g/day, which was substantially higher than the highest quartile of animal protein intake (53 g/day for males, 37 g/day for females) in our study. Alkerwi et al. [23] reported 2.5-fold higher mean animal protein intake (53.9 g/day) than ours (25.9 g/day for males and 17.4 g/day for females). Berryman et al. [8], showed negative associations between animal protein intake and obesity and the mean animal protein intake was 37.4 g/day which was lower than those from other western studies. Taken together, we carefully suggest that there may be a J-curve relationship between animal protein intake and obesity index. Below the threshold, animal protein intake might lessen obesity, and above that threshold, it might worsen obesity. More studies are needed to confirm our hypothesis.

### 4.2. Mechanism that Protein Intake Affects Obesity

Several mechanisms have been suggested to explain the observed association between protein intake and obesity. First, protein is the least efficient energy source among macronutrients using more energy in the metabolic process than carbohydrates or fats [24,25]. Second, protein increases satiety, resulting in less additional food intake [26]. The increase of peptide YY, an appetite-suppressing hormone from the gastrointestinal tract [27], and the decrease of Ghrelin, a hormone that increases appetite from the gastric parietal cells are the suggested underlying mechanisms for the satiety [28]. Cholecystokinin secreted from the duodenum by the intake of protein also suppresses appetite [29]. In addition, GLP-1 secretion induced by protein intake from the L-cell of the distal small intestine lowers the gastric emptying rate and increases satiety to suppress appetite [30]. Third, the sufficient protein intake in the elderly increases lean body mass and prevents sarcopenia, leading to an increase in both basal metabolism and physical activity, which in turn reduces obesity risk [31].

### 4.3. Renal Function and Protein Intake

One of the biggest concerns of protein intake is the possibility of impairment of renal function. Especially, since a low protein diet has been recommended to individuals with renal disease to prevent or slow the progression of renal damage. However, we found no effect of protein intake on GFR, which is in line with a recent US national study demonstrating that protein intake is not associated with a decrease in renal function in adults without chronic kidney disease [8]. Additionally, a meta-analysis of dietary intervention studies reported that high protein diet does not decrease GFR, in contrast it improves the GFR in healthy adults [32].

The RDA of protein intake for Koreans is 0.91 g/kg/day, which is lower than that of 1.0–1.2 g/kg/day by European Society for Clinical Nutrition and Metabolism (ESPEN) and European Union Geriatric Medicine Society (EUGM) [33,34]. Currently, protein intake greater than the RDA is recommended to increase muscle mass, strength and physical function in elderly with normal renal function [35]. However, 5 out of 10 Korean males and 4 out of 10 Korean females over 60 years old do not even meet the RDA [11]. Considering this, the protein intake at least up to the RDA must be encouraged for Korean elderly with normal renal function than overemphasizing the less possible risk of renal side effects.

### 4.4. Limitations and Strengths of This Study

This study has some limitations. First, the one-day 24-h recall data might have been too short to represent the usual intake of study participants. If usual intake measures were employed, the range of intake would have been reduced which may have modified associations found with one-day intake measures. Reporting bias is another limitation of the self-reported dietary data. Second, the cross-sectional study design could not infer any causal relationships between protein intake and obesity index. Third, body composition analysis like the Dual-Energy X-ray Absorptiometry was not performed; it was not possible to assess the detailed relationship between protein intake and individual components (i.e., fat, muscle, and bone) of body composition.

Despite the limitations, this study is the first in Korea to distinguish protein by its source while studying its effect on obesity. To the best of our knowledge, this study is the first in Asia.

## 5. Conclusions

In Korean adults aged 60 years or older, protein intake was associated with a favorable obesity index without decrease in renal function. The effect was similar in both male and females, with both animal and plant proteins. This outcome has potential public health implications, as promotion of a proper protein intake might attenuate obesity epidemics and subsequent cardiometabolic risks in the aged Korean population. Additional studies are warranted to explore and validate our findings.

## Figures and Tables

**Table 1:** General characteristics of the study population.

Proportion or Mean (SE) ^1^	Male	Female	Total
Unweighted, *n*	1127	1422	2549
Age (year), mean (SE)	69.5 (0.2)	69.0 (0.2)	69.2 (0.2)
60–69	54.2 (1.7)	55.9 (1.6)	55.2 (1.3)
70–79	38.8 (1.6)	36.7 (1.5)	37.6 (1.2)
80≤	6.8 (0.7)	7.2 (0.8)	7.1 (0.5)
Household income			
Lower	34.9 (1.7)	41.3 (1.8)	38.4 (1.5)
Lower middle	27.6 (1.5)	28.9 (1.5)	28.3 (1.3)
Upper middle	20.2 (1.2)	16.3 (1.2)	18.1 (1.0)
Upper	17.1 (1.4)	13.3 (1.3)	15.0 (1.2)
Education (year)			
≤9	50.2 (1.8)	78.6 (1.5)	65.8 (1.4)
10–12	29.8 (1.5)	15.1 (1.1)	21.7 (1.0)
13≤	19.8 (1.5)	6.2 (0.8)	12.3 (0.9)
Having chronic disease ^2^ vs. none	69.0 (1.5)	83.2 (1.0)	76.8 (0.9)
Body mass index, mean (SE)	23.5 (0.1)	24.4 (0.1)	24.0 (0.1)
Underweight	3.7 (0.6)	1.8 (0.4)	2.7 (0.4)
Normal	40.5 (1.6)	33.9 (1.6)	36.8 (1.1)
Overweight	25.8 (1.3)	24.7 (1.3)	25.2 (0.9)
Obese	29.8 (1.4)	39.3 (1.4)	35.0 (1.0)
Height (SE)	165.7 (0.2)	152.6 (0.2)	158.5 (0.2)
Weight (SE)	64.6 (0.3)	56.9 (0.3)	60.4 (0.2)
Waist circumference (SE)	84.8 (0.3)	82.5 (0.3)	83.5 (0.3)
Current smoker	25.2 (1.5)	2.1 (0.4)	12.5 (0.8)
Alcohol intake frequency per week			
0	28.0 (1.4)	58.3 (1.5)	44.7 (1.1)
1	38.7 (1.7)	36.3 (1.5)	37.4 (1.1)
2≤	33.1 (1.6)	5.3 (0.6)	17.8 (0.9)
MET/week (SE)	2275.1 (92.8)	1542.6 (66.3)	1872.1 (57.5)
Inactive	23.1 (1.3)	41.0 (1.6)	33.0 (1.1)
Minimally active	53.9 (1.6)	47.0 (1.5)	50.1 (1.2)
Health enhancing	22.8 (1.4)	11.8 (0.8)	16.8 (0.8)

SE, standard error; MET, metabolic equivalent for task. ^1^ Values are presented as mean or proportion (standard error) unless otherwise indicated. ^2^ Chronic diseases include hypertension, dyslipidemia, stroke, myocardial infarction, ischemic heart disease, osteoarthritis, rheumatoid arthritis, asthma, diabetes, thyroid disease, chronic kidney disease, chronic viral hepatitis, and liver cirrhosis, and any types of cancer.

**Table 2:** Energy and macronutrient intake, anthropometric characteristics, and physical activity level by the quartiles of daily total protein intake per weight of Korean elderly population.

Mean (SE) ^1^	Q1 (Lowest)	Q2	Q3	Q4 (Highest)	Total
Male					
Median, g/kg/day	0.57	0.83	1.12	1.62	
Unweighted, *n*	281	282	282	282	1127
Age, year *	70.7 (0.4)	69.7 (0.4)	68.4 (0.4)	69.1 (0.4)	69.5 (0.2)
Total energy intake, kcal/day ^†^	1406.3 (25.0)	1820.8 (25.7)	2210.1 (29.1)	2737.2 (45.2)	2033.3 (23.8)
% energy from carbohydrate, % ^†^	74.5 (0.7)	69.0 (0.8)	67.2 (0.6)	59.9 (0.9)	67.8 (0.4)
% energy from fat, % ^†^	9.9 (0.4)	12.7 (0.5)	14.8 (0.4)	18.4 (0.7)	13.9 (0.3)
Protein, total, g/day ^†^	36.3 (0.5)	54.9 (0.6)	72.2 (0.8)	107.3 (2.1)	67.1 (1.1)
% energy from protein, % ^†^	10.6 (0.2)	12.5 (0.2)	13.4 (0.2)	16.1 (0.3)	13.1 (0.1)
Protein, total, g/kg/day ^†^	0.5 (0.0)	0.8 (0.0)	1.1 (0.0)	1.8 (0.0)	1.1 (0.0)
Plant protein, g/day ^†^	28.0 (0.5)	36.9 (0.7)	45.0 (0.9)	52.7 (1.3)	40.5 (0.5)
Plant protein, g/kg/day ^†^	0.4 (0.0)	0.6 (0.0)	0.7 (0.0)	0.9 (0.0)	0.6 (0.0)
Animal protein, g/day ^†^	8.1 (0.4)	17.2 (0.8)	26.5 (1.1)	53.4 (2.5)	25.9 (0.9)
Animal protein, g/kg/day ^†^	0.1 (0.0)	0.3 (0.0)	0.4 (0.0)	0.9 (0.0)	0.4 (0.0)
A/T protein proportion, % ^†^	20.9 (1.0)	30.5 (1.2)	36.0 (1.3)	46.8 (1.4)	33.4 (0.7)
BMI, kg/m^2 †^	24.19 (0.20)	23.93 (0.21)	23.52 (0.19)	22.27 (0.20)	23.49 (0.11)
Height, cm	166.3 (0.5)	165.6 (0.3)	165.4 (0.4)	165.5 (0.4)	165.7 (0.2)
Weight, kg ^†^	67.0 (0.7)	65.7 (0.6)	64.4 (0.6)	61.1 (0.7)	64.6 (0.3)
Waist circumference, cm ^†^	87.3 (0.6)	86.3 (0.7)	84.1 (0.6)	81.5 (0.6)	84.8 (0.3)
Physical activity, MET/week	2145.8 (157.9)	2257.6 (195.9)	2218.9 (155.2)	2488.3 (181.2)	2275.1 (92.8)
Female					
Median, g/kg/day	0.46	0.70	0.95	1.46	
Unweighted, *n*	355	356	356	355	1422
Age, year ^†^	70.5 (0.4)	69.4 (0.4)	68.5 (0.4)	67.8 (0.4)	69.0 (0.2)
Total energy intake, kcal/day ^†^	1029.2 (17.6)	1394.0 (20.2)	1685.2 (22.6)	2246.2 (37.4)	1593.1 (20.1)
% energy from carbohydrate, % ^†^	78.3 (0.5)	74.9 (0.5)	72.4 (0.6)	66.6 (0.7)	73.0 (0.3)
% energy from fat, % ^†^	9.8 (0.4)	12.1 (0.4)	13.5 (0.4)	17.4 (0.5)	13.2 (0.3)
Protein, total, g/day ^†^	26.8 (0.5)	40.7 (0.4)	53.7 (0.5)	84.4 (1.4)	51.6 (0.8)
% energy from protein, % ^†^	10.6 (0.2)	12.0 (0.1)	13.2 (0.2)	15.4 (0.3)	12.8 (0.1)
Protein, total, g/kg/day ^†^	0.5 (0.0)	0.7 (0.0)	1.0 (0.0)	1.6 (0.0)	0.9 (0.0)
Plant protein, g/day ^†^	21.2 (0.4)	30.4 (0.5)	37.4 (0.7)	47.2 (1.0)	34.1 (0.5)
Plant protein, g/kg/day ^†^	0.4 (0.0)	0.5 (0.0)	0.7 (0.0)	0.9 (0.0)	0.6 (0.0)
Animal protein, g/day ^†^	5.6 (0.4)	10.2 (0.5)	16.2 (0.7)	37.0 (1.4)	17.4 (0.6)
Animal protein, g/kg/day ^†^	0.1 (0.0)	0.2 (0.0)	0.3 (0.0)	0.7 (0.0)	0.3 (0.0)
A/T protein proportion, % ^†^	19.2 (1.1)	24.5 (1.1)	29.8 (1.1)	42.0 (1.1)	29.0 (0.7)
BMI, kg/m^2 †^	25.49 (0.23)	24.83 (0.19)	24.06 (0.17)	23.30 (0.19)	24.41 (0.11)
Height, cm	152.4 (0.3)	152.6 (0.4)	152.3 (0.3)	152.9 (0.4)	152.6 (0.2)
Weight, kg ^†^	59.3 (0.6)	58.0 (0.5)	55.9 (0.4)	54.5 (0.5)	56.9 (0.3)
Waist circumference, cm ^†^	85.6 (0.6)	83.1 (0.6)	81.4 (0.5)	79.9 (0.6)	82.5 (0.3)
Physical activity, MET/week^†^	1140.3 (96.3)	1488.0 (114.3)	1666.8 (130.7)	1867.1 (168.0)	1542.6 (66.3)

SE, standard error; BMI, body mass index; Q, quartile; MET, metabolic equivalent for task; A/T protein proportion, animal total protein proportion. ^1^ Values are presented as mean (standard error) unless otherwise indicated. * *p* < 0.005, ^†^ *p* < 0.001 by trend test.

**Table 3:** Adjusted GFR and serum creatinine of kidney function by quartiles of daily total, plant, and animal protein intake per weight in Korean elderly population.

	Male (*n* = 1004)					Female (*n* = 1206)				
Quartiles	Q1	Q2	Q3	Q4	*p* for Trend	Q1	Q2	Q3	Q4	*p* for Trend
GFR, mL/min/1.73 m^2^										
Total	77.5 ± 1.1	76.7 ± 1.1	77.5 ± 1.1	79.0 ± 1.1	0.400	79.1 ± 2.1	79.1 ± 2.2	81.4 ± 2.2	80.6 ± 2.3	0.324
Plant	77.6 ± 1.1	78.2 ± 1.2	75.5 ± 1.2	75.5 ± 1.2	0.104	79.7 ± 2.0	79.1 ± 2.1	79.5 ± 2.2	82.0 ± 2.2	0.193
Animal	77.9 ± 1.3	77.3 ± 1.1	77.1 ± 1.0	77.1 ± 1.0	0.769	78.2 ± 2.1	81.3 ± 2.1	80.2 ± 2.2	80.4 ± 2.3	0.203
Creatinine, mg/dL										
Total	1.00 ± 0.02	1.01 ± 0.01	1.00 ± 0.01	0.98 ± 0.01	0.360	0.76 ± 0.02	0.76 ± 0.02	0.74 ± 0.02	0.75 ± 0.02	0.560
Plant	1.00 ± 0.02	0.99 ± 0.02	1.03 ± 0.02	0.97 ± 0.01	0.057	0.76 ± 0.02	0.77 ± 0.02	0.76 ± 0.02	0.73 ± 0.02	0.062
Animal	0.99 ± 0.02	1.00 ± 0.01	1.00 ± 0.01	0.99 ± 0.01	0.791	0.76 ± 0.02	0.74 ± 0.02	0.75 ± 0.02	0.76 ± 0.02	0.244

Q, quartile; GFR, glomerular filtration rate. Adjusted for age (year), BMI (kg/m^2^), physical activity (MET/week), household income quartiles, current smoking status (yes or no), and alcohol intake frequency per week (0, 1, 2≤). Participants were divided into quartiles for daily animal, plant, and total protein intake per weight (g/kg/day); male animal (Q1 < 0.13, Q2 0.13–0.29, Q3 0.30–0.56, Q4 0.56<), female animal (Q1 < 0.07, Q2 0.07–0.21, Q3 0.21–0.40, Q4 0.40<), male plant (Q1 < 0.45, Q2 0.45–0.61, Q3 0.62–0.79, Q4 0.79<), female plant (Q1 < 0.42, Q2 0.42–0.57, Q3 0.58–0.74, Q4 0.74<), male total (Q1 < 0.70, Q2 0.70–0.97, Q3 0.98–1.30, Q4 1.30<), female total (Q1 < 0.59, Q2 0.59–0.82, Q3 0.83–1.14, Q4 1.14<).
